# Echocardiographic parameters dynamically alter in patients with chronic kidney disease between pre- and post-dialysis

**DOI:** 10.1186/s44348-024-00005-6

**Published:** 2024-08-01

**Authors:** Dong-Hyuk Cho

**Affiliations:** Division of Cardiology, Department of Internal Medicine, Korea University Anam Hospital, Korea University College of Medicine, 73 Goryeodae-Ro, Seongbuk-Gu, Seoul, 02841 Korea

See the article “Pre- and Post-Hemodialysis Differences in Heart Failure Diagnosis by Current Heart Failure Guidelines in Patients With End-Stage Renal Disease” in volume 32.

Heart failure (HF) and chronic kidney disease (CKD) share common pathophysiological mechanisms, such as the renin–angiotensin–aldosterone system and sympathetic overactivity [1]. HF frequently coexists in patients with CKD, exacerbating both the morbidity and mortality of CKD [2]. Recently, several medications, including sodium-glucose cotransporter-2 inhibitors and finerenone, have demonstrated the ability to reduce the incidence of HF in CKD patients [3, 4]. Consequently, early detection and intervention are imperative to mitigate the development of HF in CKD patients. Current HF guidelines advocate for the utilization of echocardiographic parameters in the diagnosis of HF [5]. Specifically, multiple echocardiographic parameters, such as left ventricular mass index (LVMI), left atrial volume index (LAVI), peak tricuspid regurgitation (TR) velocity, and E/e′ ratio, are necessary to indirectly assess increased left ventricular (LV) filling pressure in patients with HF with preserved ejection fraction (HFpEF). However, the volume status of CKD patients can vary significantly based on the timing of hemodialysis. There exists a clinical unmet need to understand how hemodialysis impacts echocardiographic parameters, determine the optimal timing for conducting echocardiography, and ascertain whether the criteria for diagnosing HFpEF in CKD patients remain consistent.

In this issue of the *Journal of Cardiovascular Imaging*, Kim et al. [6] investigated how structural and functional echocardiographic parameters change in 54 chronic kidney disease patients undergoing hemodialysis, comparing echocardiographic exams taken before and after the dialysis session. This single-center study that performed echocardiography right before and after hemodialysis on the same day. While longitudinal studies exist on how echocardiographic parameters change after dialysis or transplantation, this research is important for directly comparing echocardiographic parameters before and after dialysis. The study compared echocardiographic parameters in accordance with the recent HF diagnostic criteria [5], including contemporary echocardiographic parameters like LV global longitudinal strain (GLS), not just LV EF. Body weight decreased from 62.1 kg before dialysis to 59.4 kg after, resulting in a reduction in LV end diastolic volume (LVEDV) and LV end systolic volume from 132.9 ± 41.8/104.2 ± 34.0 to 53.1 ± 28.1/39.2 ± 17.4 mL, respectively. LVMI decreased from 135 ± 46.6 to 114.9 ± 39.7 g/m^2^, while LAVI decreased from 40.6 ± 17.1 to 33.3 ± 15.9 mL/m^2^, and TR velocity decreased from 2.8 ± 0.4 to 2.5 ± 0.4 m/s. Conversely, LV EF, E/e′ ratio, and LV GLS remained unchanged. Although this study did not evaluate hard outcomes as endpoints, it analyzed echocardiographic structural and functional parameters recommended in the recent HF guidelines. The proportion meeting the guideline-recommended cut-off criteria dropped dramatically from 88.9% to 66.7% after hemodialysis.

The present study may provide insights into how echocardiographic parameters change before and after hemodialysis. It can be speculated that approximately 20% reduction in LVEDV, LAVI, and a 10% decrease in Peak TR velocity may result from around 4 h of intensive hemodialysis. Conversely, it is clinically noteworthy that these LV diastolic functional parameters will increase again before the next hemodialysis. The next question is, “How can we diagnose HF in patients with CKD?” Unfortunately, the authors were unable to answer this question due to the limitations of their study. This study was conducted with a sample size of only 54 patients at a single center. The study did not evaluate the invasive hemodynamic profile, including LV filling pressure. Therefore, it is imperative to conduct research that verifies whether the changes in echocardiographic parameters before and after hemodialysis align well with changes in invasive hemodynamics. Large-scale clinical trials employing SGLT2 inhibitors and finerenone are currently underway and are expected to greatly contribute to preventing HF in CKD patients. This study is poised to serve as an important reference point for accurately diagnosing HF in CKD patients and monitoring treatment response through echocardiography. Further studies with large sample size may offer new insights into diagnosis and treatment strategies for HF in CKD patients undergoing hemodialysis.
